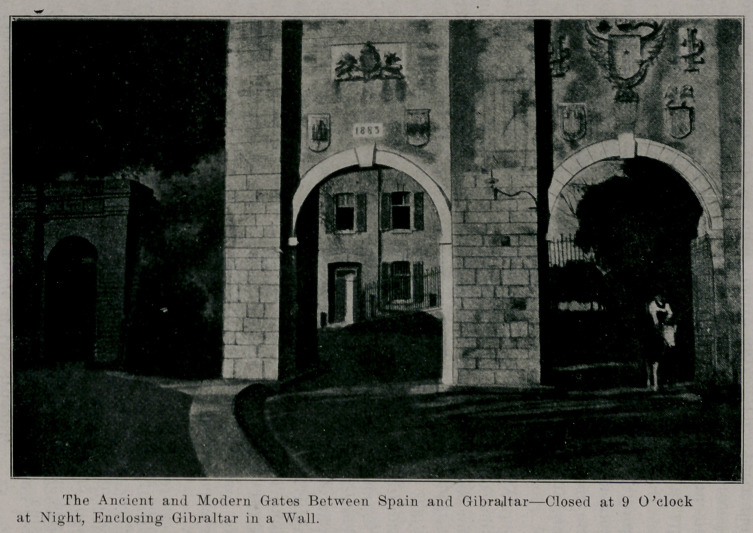# A Trip through Europe and the Hospitals

**Published:** 1914-10

**Authors:** L. C. Fischer

**Affiliations:** Atlanta, Ga.


### Journal-Record of Medicine

Successor to Atlanta Medical and Surgical Journal, Established 1855
and Southern Medical Record, Established 1870

**OWNED BY THE ATLANTA MEDICAL JOURNAL COMPANY**

PUBLISHED MONTHLY

*Official Organ Fulton County Medical Society, State Examining Board, Presbyterian Hospital, Atlanta, Birmingham and Atlantic Railroad Surgeons' Association, Chattahoochee Valley Medical and Surgical Association, Etc.*

EDGAR BALLENGER, M. D., Editor
BERNARD WOLFF, M. D., Supervising Editor
A. W. STIRLING, M. D., C. M., D. P. H.; J. S. HURT, B. Ph., M.D.
GEO. M. NILES, M. D., W. J. LOVE, M. D., (Ala.); Associate Editors
R. R. DALY, M. D., Associate Editor
E. W. ALLEN, Business Manager

**COLLABORATORS**

W. F. WESTMORELAND, M. D., General Surgery
F. W. McRAE, M. D., Abdominal Surgery
H. F. HARRIS, M. D., Pathology and Bacteriology
E. B. BLOCK, M. D., Diseases of the Nervous System
MICHAEL HOKE, M. D., Orthopedic Surgery
CYRUS W. STRICKLER, M. D., Legal Medicine and Medical Legislation
E. C. DAVIS, A. B., M. D., Obstetrics
E. G. JONES, A. B., M. D., Gynecology
R. T. DORSEY, Jr., B. S., M. D., Medicine
L. M. GAINES, A. B., M. D., Internal Medicine
GEO. C. MIZELL, M. D., Diseases of the Stomach and Intestines
L. B. CLARKE, M. D., Pediatrics
EDGAR PAULLIN, M. D., Opsonic Medicine
THEODORE TOEPEL, M. D., Mechano Therapy
A. W. STIRLING, M. D., Etc., Diseases of the Eye, Ear, Nose and Throat
BERNARD WOLFF, M. D., Diseases of the Skin
E. G. BALLENGER, M. D., Diseases of the Genito-Urinary Organs

VOL. LXI ATLANTA, GA., OCTOBER, 1914. NO. 7

### A TRIP THROUGH EUROPE AND THE HOSPITALS.

BY DR. L. C. FISCHER, ATLANTA, GA.

Now that the eyes and thoughts of the whole civilized world are centered on Europe and its doings, and too, considering the fact that for some of this time I was over there mixed up in the turmoil, think it a good time to make a casual report of the conditions, hospitals and general work as they appear to one from our side.

First, we sailed from New York early in May for Naples.

The one thing that had always been a bugbear to me about a trip across the ocean was the possibility of some severe illness aboard ship. So, as soon as we were well under way, I began to investigate the possibilities of attention should such a thing arise. Found on the lower deck a well equipped but small operating room, dispensary, sterilizing room, and all of the necessary equipment, except a capable doctor. Found that the average ship doctor is one who has not made a success at his profession and has taken this position as a last resort. When you consider that he is paid the princely sum of Sixty Dollars per month, one is not surprised at the lack of professional attainment.

With none of the alarming surgical diseases to bother me, and with a complete absence of that most feared of ocean diseases, Mal de Mere, we made a safe and pleasant journey, taking fourteen days to reach Naples.

We landed first at Gibraltar. This rock, taken from Spain in the eighteenth century by England, is one of her most precious jewels and stands today guard over the entrance and exit to the Mediterranean Sea and the far East, approached from the Atlantic.

The city has a population of twenty-six thousand, six thousand of whom are English soldiers. The sanitary conditions here are as nearly perfect as could be in a town in a temperate climate. All sanitary arrangements are directly under the English government. As they control their forts the same way, the medical men have charge of the drainage, sewerage and general sanitary conditions. I was told that they have very few diseases, but that typhoid fever was a predominating one. They have two large hospitals which I found to be immaculately clean. One is for the use of the government, that is, the soldiers and sailors; the other, while under the jurisdiction of the Governor of the Island, who is a Major General in the English Army, is for the care of the general public. The city's population is composed of English, Moors and Maltese. A small area between the two countries is declared neutral ground. No one of Gibraltar or Spain committing an offense against the law can be arrested or apprehended in any way, so long as he stays in this area. The English compel all Spaniards to leave Gibraltar by nine at night, at which time the enormous ancient gates are closed.

The forts there are perfectly wonderful. We were allowed to see a part of what is known as the "Galleries," which are some of the passages through the rock. No one is allowed to go to the top of the rock and into the forts, except an English subject.

From Gibraltar we landed at Genoa. This is an ancient city located at the extreme northern boundary of Italy on the Meditteranean. It is the principal shipping port of Italy. We were told that immense cargoes of cotton-seed oil were brought to Genoa; that this was worked over, adding a small quantity of olive oil, and returned to this country as pure imported olive oil, this being the largest olive oil market in the world. All of the mountains in Italy are covered with olive trees, a great many of which are as much as a thousand years old.

The one point of interest in Genoa was the cemetery, which has the largest Mausoleum in the southern part of Europe. In olden days all of the people were buried in the floors of the churches; but, since 1870, when Italy became a united country, they have used the mausoleums for burial grounds. The poor are buried in a cemetery which is surrounded by this enormous mausoleum. They are allowed to occupy a grave for five years, at the end of which time the body is exhumed and cremated, the ashes being turned over to the family, where desired. Under no circumstances is a poor person allowed to occupy a grave longer than five years.

From Genoa we went to Naples. Upon landing, we were immediately impressed with the poverty, filth, ignorance, and the general depravity of the people. Never have I seen such unsanitary surroundings and such intense poverty. It was practically impossible to cross the streets or leave your hotel door without being approached by some beggar, who was in a great many cases deformed, or had lost a leg, an arm or an eye in some of the many strikes or revolts against the government rule that these people have.

Visited one of the ancients hospitals, but only for a few moments. Conditions as a whole were so uninteresting that did not investigate further. The building was old and delapidated; the rooms and the halls very dark; all the windows shut tight and the blinds closed. Was much surprised to find in this balmy climate that the people did not open their windows for fresh air, but kept the doors and windows closed practically night and day.

Was agreeably surprised to find the absence of mosquitoes. Can not say the same for fleas. It seemed to me at the time that it would be necessary for one to surround himself with water or antiseptic in order to escape the ravages of this little black pest. The general unsanitary conditions are impossible to describe. My poverty of adjectives leaves me at a loss to say other than that they are unbelievable. We found in this city of Naples livery stables run on the ground floor, and the people in unbelievable numbers living on the floor above and often in stables with horses.

The houses and hospitals are built in all cases of fireproof material, which is cement blocks, this being largely necessary on account of the scarcity of lumber—and too, these people build for generations, each house is so constructed that it will stand for centuries, while we build for today. The streets are paved with lava stones, which are from two to three feet thick, making a pavement that will last for hundreds of years.

A bath tub we found to be a luxury; in fact, we were convinced that the Italians only used water to row boats over. They drink native wines, which contain about two to three per cent alcohol.

The diseases they have to fear are cholera, bubonic plague, and what they call slow fever. Syphilis and tuberculosis are predominating conditions, cancers are also much more frequent than in the states; all kinds of Genito-Urinary diseases are very prevalent. The people look old and poorly kept while still

MILITARY AND CIVILIAN HOSPITALS AT GIBRALTAR

young. The women especially show the results of the hardships to which they are subjected; they break early; and in most cases they raise large families. Practically all the people are brunettes. The women and the small donkeys are the beasts of burden. It seemed cruel for the women to carry the loads that we saw them laboring under. A great many Caesarian sections are done on account of the deformities from labor and syphilis—then, too, so many young girls are impregnated before they are well enough developed to give birth to a child. Most of the heavy burdens are carried on the head, but in a great many cases they have it strapped on the back. In many instances we saw old women bent and bowed from the impossible weights carried. A great many of the children are suffering from Rickets and the results, general inanition is a predominating condition.

The people are universally lazy; often while on drives we would see men asleep, lying on the side walks or in the roads.

On a drive into the rural districts from Naples, we passed numerous homes where a jack, chickens, a cow and the people were all living in one room. When a rain came up, the cart was rolled in to add to their many discomforts. We were told that it was useless to take a bath in Italy, as at some future times, within two or three months, it would be necessary to take another.

At the National Museum in Naples we saw many of the old and original instruments that have been found during the excavations at Pompeii. It is known from scientific investigations that Pompeii and Herculaneum existed four hundred years before Christ. It is also known that the city was destroyed in the year 79 A. D. It was not until the seventeenth century that the excavations were begun there. During the time that the work has gone on, various surgical instruments have been found and preserved by the Italian government. These are displayed with many relics of ancient mythology. There is certainly no myth connected with the various instruments, which, in a great many instances, are of the same and better design than those used today. There is, for instance, one saw for amputations which I believe is large enough to have amputated the extremities of that great giant of Roman mythology, Hercules. Even though he is depicted as bearing the world on his shoulders and doing other tasks equally as great, the saw certainly could have reached through the thigh. Then, too, the gall bladder instruments, scoops, probes ladles and such things are practically the same design as we are now using. The one most notable instrument was a rectal dilator. This was made to do its work very gradually, dilating with a small lever and set screws. It was made with four blades instead of two. The knives were very much the same design as we now use, except that the handles were wide and much heavier than ours. The bone instruments were numerous, chisels, curetts, probes and such things as are familiarly found in our instrument shops. An instrument that I thought was a needle-holder was marked "dental forceps." The hemostatts looked more like tissue forceps than the instruments they were designed for. But when I reached Germany, I found that the surgeons were using the same designs as the old ones. The vaginal specula were cylindrical, bivalve and trivalve. Then, too, there were instruments which were too much disfigured by the fire to recognize their use.

One of the most interesting things to be seen at Naples is Mt. Vesuvius. We went up to the crater and looked down into the veritable hell that it is. If there is anything on this earth that will increase our fear of that hell we are taught something of, it is that great boiling volcano. Constantly it is emitting great billows of sulphur fumes with blazes of fire licking up amidst the smoke. This is the way the volcano was behaving just before the ancient city of Pompeii was destroyed. It was said that the mountain is much more active for the last six months that it has been for years; in fact, since 1906, when a small city at the base of the mountain was destroyed, killing about six hundred people. Even with the remains of Pompeii and Herculaneum being constantly excavated, they are building a new city of Pompeii directly at the foot of this old destroyer of people and property. There are several small towns that stand around its base, some of them having stood there for centuries. In all cases they bear the name of some martyred saint. In many of the streets are shrines for worship. They hope in this way to have the Virgin watch over them and to keep their city from being destroyed by the volcano.

Most of the people of Italy are Roman Catholics, except those who compose the new political party, who choose to call themselves Socialists. They have in all cases denounced the church and work against the government.

The mode of transportation is the same as existed in the days of Christ—a small jack or an ox hitched to a two-wheeled cart. They work the milch cows to the wagons, in this way saving the expense of keeping an ox. The principal industries are farming, raising fruit and making wines, hay and grain. We saw the most wonderful fields of these. We were told not to eat the fruits nor to drink the water of the country. But the fruit was so tempting that we ate, and then, after seeing how filthy the people were and how they shunned water, we decided this was much better than what they would bottle, and that we could and did drink freely to no bad results. We tried the native wines, and after one sample, were satisfied to abstain all the way through Europe. Manufacturing of macaroni and spaghetti is one of their main sources of revenue. A body only needs to see one macaroni factory to be perfectly satisfied for the balance of his life with what they have eaten, and to allow their enemies to eat the balance.

From Naples we visited the ancient and destroyed city of Pompeii. There are many things which remain and which are pointed out by the guide that convince one that Pompeii was really destroyed on account of its great immorality.

The volcanic eruption of Mt. Vesuvius was supposed to have taken place in August, when many of the people were away in the mountains. This is judged from the fact that so few bodies were found and are now being found in the excavations. When working, they come to an opening in the ground or in the lava, this they fill with plaster of paris, and when taken out, in a great many cases, is found to be the form of a human being, dogs, hogs, etc. Many of the forms show the great agony that they experienced when death came. I was especially impressed with the figure of a woman which the

The Ancient and Modern Gates Between Spain and Gibraltar—Closed at 9 O'clock at Night, Enclosing Gibraltar in a Wall.

guide explained was about seven months pregnant when she was burned. From the general contour of the body, and especially of the abdomen, this was believable. On some of the walls were painted the serpent as representing a doctor's home or office, also the mortar and pestle of the druggist.

From Naples we went to what is called New Rome. This seemed to me a very inappropriate name for the ancient and forgotten remains of what is almost prehistoric. From the time we reached Rome until the day we left, we lived in the beginning of the first and second centuries. Once or twice do I faintly recall that our guide told us that this or that building was destroyed in the third or fourth centuries.

We were shown a hospital which is on St. Bartholomew's Island in the middle of the Tiber river, which was said to have been built 290 years before Christ. Some few years ago the enterprising builders of the States thought that they had made quite a discovery when they began building with Ferro-Concrete. They would be surprised to go to Rome and find that this old hospital, still standing and now being used as a hospital, with one hundred beds, is built of Ferro-Concrete. There is a bridge across the portion of the River Tiber leading to the island which was said to have been built 200 years before Christ. This is still in use. The hospital, it was explained to us by our guide, has been modernized—certain improvements were made on it during the 7th and 8th centuries. Since then it has run and still is in practically the same condition.

Most of the poor people, when sick, are cared for by the numerous charitable organizations and insurance companies. Every person is required to carry some form of industrial insurance, all of them for sickness, and those who can afford it, for death.

The second day we were there, there was a strike of all of the Socialists, this being a protest against the government for the gendarmes having killed two Socialists, two days preceding our visit on one of their festive days. This particular festive day was in commemoration of the death of a martyred saint whose bones are still kept as sacred, and which are displayed to a worshipping congregation or population once each year.

It was blood-curdling to go to one of the old churches and find the skeleton of some individual, be he saint or sinner, preserved in a glass coffin, and this placed inside of a glass vault, which was most sacred. The bones, as a rule, are decorated with many costly jewels, the skeleton having glass eyes and oftentimes jewels or gold in the teeth. The bones are covered with all kinds of filigree. It is only necessary to expose the skeleton and have the people come into full view of it, for them to receive a blessing that will last for the next twelve months.

The strike, as it was called, was not for higher wages, or shorter hours, but simply a protest against the government. We found that all of the working classes were members of this Socialistic party, which included the street car and railroad men, postal authorities, cab and hack drivers and the majority of the railroad people. For four days we were unable to secure any kind of transportation. While out one day walking the shooting was so dangerously near that we very much feared for our safety as there was only a brick wall separating us from the firing. We were told that as a result of one day's rioting there were fifty men killed and wounded, one of whom was the Assistant Chief of Police.

I was perfectly horrified to find the wounded being rushed to the hospitals on small push cart ambulances. These were built on two wheels and were propelled by two very insignificant looking soldiers, the patient being covered over with a kind of tent. Found these same ambulances, which in many cases are owned by the insurance companies, being used to transport the sick, especially the poorer classes.

While in Rome, visited the Royal Hospital, and saw there my friend, Dr. Raphael Bastianelli doing some of the most wonderful surgery that it has ever been my pleasure to see. His operating table was of a design far superior to anything ever seen. It was the result of his and other surgeons' efforts and was built in Turin, Italy. With foot levers he was enabled to get any position desired. Dr. Bastianelli is surgeon to the Royalty of Italy, and, while apparently a young man, is one of most unusual ability and literary attainments. He speaks English well and extends to any visiting surgeon a royal welcome. His instruments, technique, etc., were in keeping with the best. A great deal of his work was done under local anaesthesia. I saw him remove goiters, trephines, and do operations upon the stomach, together with the removal of the appendix with local and spinal anaesthesia. He stated to me that his results in spinal anaesthesia were good. But, I noticed in a great many instances that while the patients were strapped to the table and were under the absolute control of the operator, they were constantly cringing from pain. All patients are strapped to the table, the hands first, then a broad strap over the legs and one over the chest or abdomen, according to the site of operations. His work was done in almost bloodless fields. The slightest bleeding point was caught and ligated. His method of cutting across muscles was especially instructive; before cutting he would sew two lines of sutures through the muscles, tie them, and cut between, when the ends of the cut muscle would stand up in a bundle and not retract back into its sheath or fascia. Saw him cut the right rectus in a man to remove a large Recto-peritoneal Lipoma. When the wound was ready to close the ends of the muscle approximated beautifully. Practically all of his sewing was interrupted sutures. While he is a very famous surgeon, he is on duty for twenty-four hours every third day. There are three chief surgeons, each one having to follow this routine. The hospital has more than 1200 beds which are kept up by the government. Was especially interested in two cases where the abdomen was opened for exploration. In each case the patient complained of pain and suffered greatly with indigestion. He finally removed the appendix from each. Recently I had removed a great many chronic appendices for indigestion and general abdominal pains where the patients had experienced great relief. This we discussed; he stated he would not be willing to express an opinion as to whether the operation was beneficial or not under one year. A letter from Dr. Bastianelli two months later stated that "the patients are still well but can't tell of their real condition under a year." I mention this only to show the care and investigation of European surgeons. Was told by a medical man in Rome that as the result of the use of spinal anaesthesia, he, the medical man, was kept constantly busy; that the patients suffered for months with headache, pains in the back and extremities, that often there was partial loss of locomotion, in many instances a complete loss of control of the functions of the bowels and kidneys, and in numerous cases a complete loss of sexual powers. This was explained not to mean much with women, but was a serious thing with men.

The people in the clinic were suffering with various diseases—a great many cancers, tuberculosis, and all genito-urinary diseases. Flat-foot was a predominating condition.

The hospital is built on the pavilion plan and is composed of forty-eight buildings, which cost the government $3,800,000. They are still building, and have under construction now two modern buildings for orthopedic work and a pathological laboratory. The buildings are of fire-proof material and are from one to four stories high, connected by cement walk ways, which are all covered over. The halls are for the most part very narrow and dark. The rooms are meagerly furnished, but neatly kept. The operating rooms are small and not the most modern, the walls being plain plaster. All the gas and water pipes are exposed. The light in the operating room was not as we are used to. But, with these conditions, the work was as good, or possibly surpassed, anything I saw in Europe. Found Dr. Bastianelli not rapid, but most accurate. His diagnoses were to me a revelation from the standpoint of accuracy. Found that he used all of the modern operating room appliances—gloves, masks, etc., and that he used exclusively silk and linen. Saw very little catgut used during my entire trip on the continent. Was surprised to find that he had in this hospital a number of English women for the operating room and head nurses. In fact, the conduct of this hospital, its general appearance, etc., impressed one with the ability of the one at its head or one of its chief advisors.

The poverty of Rome impressed me as being as bad, if not worse than that of Naples. Never did we enter one of the ancient churches, but that we were besieged both at entrance and exit by a band of poverty-striken, woe-begone individuals. It was useless to try to give to one because it would simply add to the number who would follow you.

The churches of Rome are the remains of what at one time was the pride of the Roman Catholic church of the world. None of them have seats or benches for services. A great majority of the people that we saw in them were either beggars or the unemployed. We were shown in one of them what is known as the "Holy Stairway." This was supposed to have been the stairway that lead to the court of Pilate, and up and down which Christ went when He was being tried by this high magistrate. It is positively prohibited to come down these stairs at any cost, and you only ascend them on your knees. Was impressed with the people, except some tourists, who were making this ascension. They were without an exception the poverty-stricken of the poor. Was also impressed with a pamphlet which was given out that had a printed prayer to be made to the Virgin on each step. Martin Luther was the only man in ancient times who was brave enough to walk down these stairs and denounce the Roman Catholic church. Under no circumstances is his name mentioned by a true Roman Catholic. We were also shown in another old church a place where the chains that bound St. Peter are kept; also in the Church of St. John Lateron the place where the cradle of Christ is kept, and then the holiest of all, the table whereon He ate the Last Supper.

We were also carried through what is known as the catacombs. These consist of a series of passageways dug some twenty feet under the ground. The graves or vaults were dug in the sides of the wall and were rented out to the people by the church or by the priests who owned them. They were first used to bury some of the old apostles. Our guide, who was a very learned man, pointed out to us the exact one in which St. Paul and St. Peter were first interred. The Roman Catholics, in after years, robbed these vaults, moving all of the bones to the sites of their churches and new buildings, thinking that only saints' bones were buried there. Some of these shelves still contain the bones of persons buried some eighteen or nineteen hundred years ago. It is needless to say that the odor was unbearable, and, too, it would be needless to add, that the water in the vicinity of Rome is considered to be of no use. The water supply comes to the city from the mountains some seventy miles away through aqueducts, some of which are standing that stood in the days of Christ.

A part of the old Roman wall still stands and especially the gate to the city on the Appian Way. This old arch is pointed out as being the one through which Saint Paul passed on his entrance into the city when he resided there for two years, and through which Saint Peter went when he was leaving Rome and when Christ met him on the Appian Way and sent him back into Rome to be crucified, which history tells us was done with his head down.

We were shown in the Church of St. Sebastian some foot prints of Christ, which are about four inches deep in solid stone. These are said to be the result of Christ's standing on this stone talking to St. Peter and rebuking him for his attempt to escape martyrdom. Even though Christ has been crucified for a number of years, they explain to you with the greatest confidence that these are the original footprints. We were told that there were in all four sets of the footprints to be seen in the city. It struck me as being rather a ridiculous thing. From examination we believed that they were both the moulds of the right foot. It is useless to say that many of the things told us were unbelievable. We saw the exact places in the Roman Forum where Caesar was stabbed, and where, afterwards, Mark Anthony made his wonderful oration over the remains. Though these things happened centuries ago, the places are still pointed out accurately. On the Palatine Hill we were shown the exact room in the old destroyed palace of the crazy King Caligula, where gilded beans were fed to his favorite horse; also the room in which his guests were beheaded in the event they refused to dine with the horse or partake of his provender. Adjoining his dining room, which was next to his bed room, there was an open chute, where after eating a full meal he would go and lean over, tickle his throat with a feather, vomit up the meal, and then eat another just as large and satisfying.

One is impressed with the wonderful building ability of the ancient Romans. Many of the old walls, the buildings of which have been destroyed for centuries, are still standing, and show that in their day they were built by architects and builders such as we have not.

The bath houses, which were public, were so built that steam or dry heat passed between two cement walls and kept the buildings comfortable.

One could really profit by reading again the "Last Days of Pompeii," as some of its descriptions are very accurate.

(To Be Continued.)

## Figures and Tables

**Figure f1:**
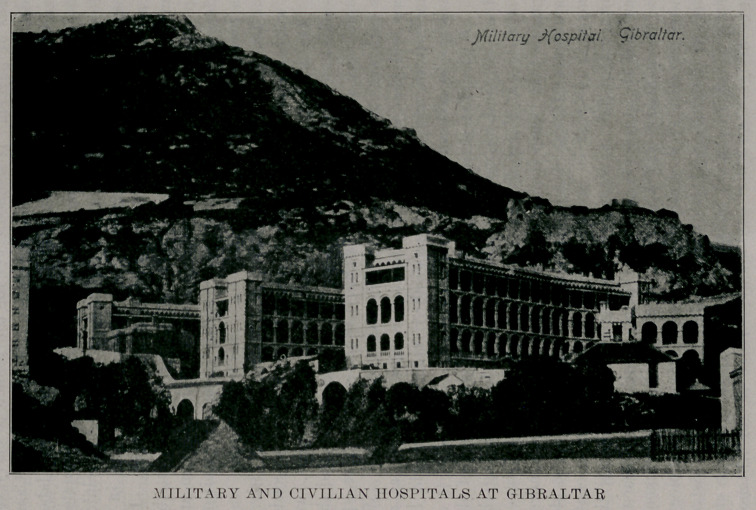


**Figure f2:**